# Supplementary material for: Comprehensive Comparative Analyses of Aspidistra Chloroplast Genomes: Insights into Interspecific Plastid Diversity and Phylogeny
Source: Genes (Basel). 2023 Sep 29;14(10):1894. doi: 10.3390/genes14101894 (PMC10606303; doi:10.3390/genes14101894)
Supplement: Supplementary file 1 [file genes-14-01894-s001.zip › genes-2604441-supplementary.pdf]

Table S1. Information of the seven newly sequenced *Aspidistra* taxa in this study.

Species	Collector	Collection places	Coverage depth	Voucher information	Herbarium information
Aspidistra minutiflora	Chunrui Lin	Haokun Village, Lingyun County, Guangxi Zhuang Autonomous Region, China	63	1072	IBK
Aspidistra retusa	Chunrui Lin	Xingping Town, Yangshuo County, Guilin City, Guangxi Zhuang Autonomous Region, China	236	1104	IBK
Aspidistra dolichanthera	Chunrui Lin and Weibin Xu	Longzhou County, Guangxi Zhuang Autonomous Region, China	178	1309	IBK
Aspidistra crassifila	Chunrui Lin	Dongzhong Town, Fangchenggang City, Guangxi Zhuang Autonomous Region, China	147	10013	IBK
Aspidistra erecta	Chunrui Lin	Jingxi City, Guangxi Zhuang Autonomous Region, China	179	8445	IBK
Aspidistra longgangensis	Chunrui Lin	Longzhou County, Guangxi Zhuang Autonomous Region, China	501	1359	IBK
Aspidistra nankunshanensis	Chunrui Lin	Nankun Mountain, Longmen County, Guangdong Province, China	341	1107	IBK

Table S2. Gene composition in the Aspidistra plastid genomes.

Category of Genes	Group of Genes	Name of Genes
Self-replication	Ribosomal RNA	*rrn*16S (x2), *rrn*23S (x2), *rrn*5S (x2), *rrn4.*5S (x2)
	Transfer RNA	*trnA*-UGC*(x2), *trnC*-GCA, *trnD*-GUC, *trnE*-UUC, *trnF*-GAA, *trnG*-GCC, *trnG*-UCC*, *trnH*-GUG (x2), *trnI*-CAU (x2), *trnI*-GAU*(x2), *trnK*-UUU*, *trnL*-CAA (x2), *trnL*-UAA*, *trnL*-UAG, *trnM*-CAU, *trnN*-GUU (x2), *trnP*-UGG, *trnQ*-UUG, *trnR*-ACG (x2), *trnR*-UCU, *trnS*-GCU, *trnS*-GGA, *trnS*-UGA, *trnT*-GGU, *trnT*-UGU, *trnV*-GAC (x2), *trnV*-UAC*, *trnW*-CCA, *trnY*-GUA, *trnfM*-CAU
	Large subunit of ribosome	*rpl14*, *rpl16**, *rpl2**(x2), *rpl20*, *rpl22*, *rpl23* (x2), *rpl32*, *rpl33*, *rpl36*
	DNA dependent RNA polymerase	*rpoA*, *rpoB*, *rpoC1**, *rpoC2*
	Small subunit of ribosome	*rps11*, *rps12**(x2), *rps14*, *rps15*, *rps16**, *rps18*, *rps19* (x2), *rps2*, *rps3*, *rps4*, *rps7* (x2), *rps8*
Photosynthesis	Subunits of ATP synthase	*atpA*, *atpB*, *atpE*, *atpF**, *atpH*, *atpI*
	Subunits of photosystem II	*psbA*, *psbB*, *psbC*, *psbD*, *psbE*, *psbF*, *psbH*, *psbI*, *psbJ*, *psbK*, *psbL*, *psbM*, *psbN*, *psbT*, *psbZ*, *ycf3***
	Subunits of NADH-dehydrogenase	*ndhA**, *ndhB**(x2), *ndhC*, *ndhD*, *ndhE*, *ndhF*, *ndhG*, *ndhH*, *ndhI*, *ndhJ*, *ndhK*
	Subunits of cytochrome b/f complex	*petA*, *petB**, *petD**, *petG*, *petL*, *petN*
	Subunits of photosystem I	*psaA*, *psaB*, *psaC*, *psaI*, *psaJ*
	Subunit of rubisco	*rbcL*
Other genes	Subunit of Acetyl-CoA-carboxylase	*accD*
	c-type cytochrome synthesis gene	*ccsA*
	Envelop membrane protein	*cemA*
	Protease	*clpP***
	Maturase	*infA*
Unknown	Conserves open reading frames	*ycf*1, *ycf*2 (x2), *ycf*4

Note: (x2) indicates that the gene is located in the IR regions, resulting in two copies; * and ** represent genes with one/two introns, respectively.

Table S3. Simple sequence repeats (SSRs) in the seven *Aspidistra* plastid genomes.

ID	SSR nr.	SSR type	SSR	size	start	end
Aspidistra minutiflora	1	p1	(A)11	11	2942	2952
Aspidistra minutiflora	2	p1	(A)10	10	3563	3572
Aspidistra minutiflora	3	p1	(A)10	10	3825	3834
Aspidistra minutiflora	4	p4	(TAAT)3	12	4450	4461
Aspidistra minutiflora	5	p1	(A)10	10	4538	4547
Aspidistra minutiflora	6	p3	(TTA)4	12	4648	4659
Aspidistra minutiflora	7	p1	(C)10	10	4924	4933
Aspidistra minutiflora	8	p1	(T)10	10	7994	8003
Aspidistra minutiflora	9	p2	(TA)8	16	9223	9238
Aspidistra minutiflora	10	p2	(AT)5	10	14670	14679
Aspidistra minutiflora	11	p3	(TTA)4	12	15730	15741
Aspidistra minutiflora	12	p1	(T)15	15	16486	16500
Aspidistra minutiflora	13	p1	(T)10	10	18547	18556
Aspidistra minutiflora	14	p1	(T)11	11	18653	18663
Aspidistra minutiflora	15	p2	(AT)5	10	20032	20041
Aspidistra minutiflora	16	p1	(A)10	10	22708	22717
Aspidistra minutiflora	17	p1	(T)10	10	23099	23108
Aspidistra minutiflora	18	p1	(A)11	11	27879	27889
Aspidistra minutiflora	19	p1	(T)10	10	28484	28493
Aspidistra minutiflora	20	p3	(ATA)4	12	30037	30048
Aspidistra minutiflora	21	p2	(TA)5	10	30083	30092
Aspidistra minutiflora	22	p1	(A)15	15	44327	44341
Aspidistra minutiflora	23	p5	(TTACA)3	15	46713	46727
Aspidistra minutiflora	24	p1	(A)14	14	47054	47067
Aspidistra minutiflora	25	p2	(AT)5	10	47133	47142
Aspidistra minutiflora	26	p1	(A)12	12	52347	52358
Aspidistra minutiflora	27	p2	(TA)5	10	55693	55702
Aspidistra minutiflora	28	p4	(ATTG)3	12	57585	57596
Aspidistra minutiflora	29	c	(AT)5ca(AT)5	22	57998	58019
Aspidistra minutiflora	30	p2	(TC)5	10	62040	62049
Aspidistra minutiflora	31	p4	(AATG)3	12	62659	62670
Aspidistra minutiflora	32	p2	(TA)5	10	68663	68672
Aspidistra minutiflora	33	p2	(AT)8	16	69104	69119
Aspidistra minutiflora	34	p1	(T)10	10	69669	69678
Aspidistra minutiflora	35	p1	(T)10	10	72009	72018
Aspidistra minutiflora	36	p1	(A)10	10	72504	72513
Aspidistra minutiflora	37	p1	(A)10	10	72537	72546
Aspidistra minutiflora	38	p1	(T)10	10	75197	75206
Aspidistra minutiflora	39	p1	(T)10	10	83051	83060
Aspidistra minutiflora	40	p4	(AATA)3	12	83098	83109
Aspidistra minutiflora	41	p2	(GA)5	10	90921	90930
Aspidistra minutiflora	42	p1	(T)10	10	101384	101393
Aspidistra minutiflora	43	p4	(GAAT)3	12	105737	105748
Aspidistra minutiflora	44	p5	(CGAAA)3	15	109624	109638
Aspidistra minutiflora	45	p1	(A)11	11	114003	114013
Aspidistra minutiflora	46	p1	(A)10	10	114141	114150

ID	SSR nr.	SSR type	SSR	size	start	end
Aspidistra minutiflora	47	p1	(T)10	10	114541	114550
Aspidistra minutiflora	48	p4	(AATT)3	12	115171	115182
Aspidistra minutiflora	49	p3	(ATT)4	12	115629	115640
Aspidistra minutiflora	50	p1	(A)11	11	116389	116399
Aspidistra minutiflora	51	p4	(AATA)3	12	117212	117223
Aspidistra minutiflora	52	p4	(TTGA)3	12	119306	119317
Aspidistra minutiflora	53	p1	(T)11	11	120382	120392
Aspidistra minutiflora	54	p1	(A)10	10	122353	122362
Aspidistra minutiflora	55	p3	(ATT)4	12	125113	125124
Aspidistra minutiflora	56	p1	(T)10	10	126271	126280
Aspidistra minutiflora	57	p1	(A)10	10	128705	128714
Aspidistra minutiflora	58	p5	(TTTCG)3	15	131883	131897
Aspidistra minutiflora	59	p4	(CATT)3	12	135772	135783
Aspidistra minutiflora	60	p1	(A)10	10	140128	140137
Aspidistra minutiflora	61	p2	(TC)5	10	150591	150600
Aspidistra retusa	1	p1	(A)11	11	2942	2952
Aspidistra retusa	2	p1	(A)12	12	3563	3574
Aspidistra retusa	3	p4	(TAAT)3	12	4451	4462
Aspidistra retusa	4	p1	(A)11	11	4539	4549
Aspidistra retusa	5	p3	(TTA)4	12	4650	4661
Aspidistra retusa	6	p1	(C)11	11	4926	4936
Aspidistra retusa	7	p2	(TA)8	16	9214	9229
Aspidistra retusa	8	p2	(AT)5	10	14659	14668
Aspidistra retusa	9	p3	(TTA)4	12	15719	15730
Aspidistra retusa	10	p1	(T)15	15	16475	16489
Aspidistra retusa	11	p1	(T)10	10	18543	18552
Aspidistra retusa	12	p1	(T)11	11	18649	18659
Aspidistra retusa	13	p2	(AT)5	10	20028	20037
Aspidistra retusa	14	p1	(A)11	11	22704	22714
Aspidistra retusa	15	p1	(A)11	11	28803	28813
Aspidistra retusa	16	p3	(ATA)4	12	30034	30045
Aspidistra retusa	17	p2	(TA)5	10	30080	30089
Aspidistra retusa	18	p1	(A)15	15	44389	44403
Aspidistra retusa	19	p1	(A)12	12	47138	47149
Aspidistra retusa	20	p2	(AT)5	10	47215	47224
Aspidistra retusa	21	p1	(T)10	10	48120	48129
Aspidistra retusa	22	p1	(A)12	12	52401	52412
Aspidistra retusa	23	p2	(TA)5	10	55747	55756
Aspidistra retusa	24	p4	(ATTG)3	12	57639	57650
Aspidistra retusa	25	p2	(AT)5	10	58052	58061
Aspidistra retusa	26	p2	(TC)5	10	62093	62102
Aspidistra retusa	27	p4	(AATG)3	12	62712	62723
Aspidistra retusa	28	p2	(TA)5	10	68622	68631
Aspidistra retusa	29	p2	(AT)9	18	69073	69090
Aspidistra retusa	30	p1	(T)11	11	69640	69650
Aspidistra retusa	31	p1	(A)10	10	71455	71464
Aspidistra retusa	32	p1	(T)10	10	71710	71719
Aspidistra retusa	33	p1	(T)10	10	71983	71992

ID	SSR nr.	SSR type	SSR	size	start	end
Aspidistra retusa	34	p1	(A)10	10	72478	72487
Aspidistra retusa	35	p1	(T)12	12	79336	79347
Aspidistra retusa	36	p4	(AATA)3	12	83083	83094
Aspidistra retusa	37	p2	(GA)5	10	90910	90919
Aspidistra retusa	38	p1	(T)10	10	101368	101377
Aspidistra retusa	39	p4	(GAAT)3	12	105721	105732
Aspidistra retusa	40	p5	(CGAAA)3	15	109605	109619
Aspidistra retusa	41	p1	(A)10	10	113984	113993
Aspidistra retusa	42	p1	(C)12	12	114284	114295
Aspidistra retusa	43	p1	(T)10	10	114532	114541
Aspidistra retusa	44	p4	(AATT)3	12	115161	115172
Aspidistra retusa	45	p1	(T)10	10	115265	115274
Aspidistra retusa	46	p3	(ATT)4	12	115625	115636
Aspidistra retusa	47	p1	(A)11	11	116385	116395
Aspidistra retusa	48	p4	(AATA)3	12	117208	117219
Aspidistra retusa	49	p4	(TTGA)3	12	119302	119313
Aspidistra retusa	50	p1	(A)12	12	122341	122352
Aspidistra retusa	51	p3	(ATT)4	12	125103	125114
Aspidistra retusa	52	p1	(T)11	11	126241	126251
Aspidistra retusa	53	p1	(A)10	10	128675	128684
Aspidistra retusa	54	p5	(TTTCG)3	15	131838	131852
Aspidistra retusa	55	p4	(CATT)3	12	135724	135735
Aspidistra retusa	56	p1	(A)10	10	140080	140089
Aspidistra retusa	57	p2	(TC)5	10	150538	150547
Aspidistra dolichanthera	1	p1	(A)11	11	2936	2946
Aspidistra dolichanthera	2	p1	(A)17	17	3557	3573
Aspidistra dolichanthera	3	p4	(TAAT)3	12	4450	4461
Aspidistra dolichanthera	4	p1	(A)11	11	4538	4548
Aspidistra dolichanthera	5	p3	(TTA)4	12	4649	4660
Aspidistra dolichanthera	6	p1	(C)15	15	4925	4939
Aspidistra dolichanthera	7	p2	(TA)9	18	9191	9208
Aspidistra dolichanthera	8	p2	(AT)5	10	14641	14650
Aspidistra dolichanthera	9	p3	(TTA)4	12	15701	15712
Aspidistra dolichanthera	10	p1	(T)15	15	16457	16471
Aspidistra dolichanthera	11	p1	(T)10	10	18525	18534
Aspidistra dolichanthera	12	p1	(T)11	11	18631	18641
Aspidistra dolichanthera	13	p2	(AT)5	10	20010	20019
Aspidistra dolichanthera	14	p1	(A)11	11	22686	22696
Aspidistra dolichanthera	15	p1	(A)10	10	27851	27860
Aspidistra dolichanthera	16	p3	(ATA)4	12	30008	30019
Aspidistra dolichanthera	17	p2	(TA)5	10	30054	30063
Aspidistra dolichanthera	18	p1	(A)14	14	44348	44361
Aspidistra dolichanthera	19	p1	(A)14	14	47081	47094
Aspidistra dolichanthera	20	p2	(AT)5	10	47160	47169
Aspidistra dolichanthera	21	p1	(T)11	11	48065	48075
Aspidistra dolichanthera	22	p1	(A)12	12	52348	52359
Aspidistra dolichanthera	23	p2	(TA)5	10	55694	55703
Aspidistra dolichanthera	24	p4	(ATTG)3	12	57586	57597

ID	SSR nr.	SSR type	SSR	size	start	end
Aspidistra dolichanthera	25	c	(AT)5ca(AT)5	22	57999	58020
Aspidistra dolichanthera	26	p2	(TC)5	10	62041	62050
Aspidistra dolichanthera	27	p4	(AATG)3	12	62660	62671
Aspidistra dolichanthera	28	p2	(TA)5	10	68633	68642
Aspidistra dolichanthera	29	p2	(AT)6	12	69084	69095
Aspidistra dolichanthera	30	p1	(T)11	11	69645	69655
Aspidistra dolichanthera	31	p1	(A)11	11	71460	71470
Aspidistra dolichanthera	32	p1	(T)12	12	71716	71727
Aspidistra dolichanthera	33	p1	(A)11	11	72485	72495
Aspidistra dolichanthera	34	p1	(T)10	10	75178	75187
Aspidistra dolichanthera	35	p1	(T)10	10	83047	83056
Aspidistra dolichanthera	36	p4	(AATA)3	12	83094	83105
Aspidistra dolichanthera	37	p2	(GA)5	10	90915	90924
Aspidistra dolichanthera	38	p1	(T)10	10	101378	101387
Aspidistra dolichanthera	39	p4	(GAAT)3	12	105731	105742
Aspidistra dolichanthera	40	p5	(CGAAA)3	15	109615	109629
Aspidistra dolichanthera	41	p1	(A)10	10	113818	113827
Aspidistra dolichanthera	42	p1	(A)10	10	113995	114004
Aspidistra dolichanthera	43	p1	(A)11	11	114132	114142
Aspidistra dolichanthera	44	p1	(C)11	11	114297	114307
Aspidistra dolichanthera	45	p1	(T)10	10	114535	114544
Aspidistra dolichanthera	46	p4	(AATT)3	12	115165	115176
Aspidistra dolichanthera	47	p3	(ATT)4	12	115629	115640
Aspidistra dolichanthera	48	p1	(A)11	11	116389	116399
Aspidistra dolichanthera	49	p4	(AATA)3	12	117212	117223
Aspidistra dolichanthera	50	p4	(TTGA)3	12	119306	119317
Aspidistra dolichanthera	51	p1	(T)12	12	120382	120393
Aspidistra dolichanthera	52	p1	(A)10	10	122348	122357
Aspidistra dolichanthera	53	p3	(ATT)4	12	125108	125119
Aspidistra dolichanthera	54	p1	(T)10	10	126246	126255
Aspidistra dolichanthera	55	p1	(A)10	10	128680	128689
Aspidistra dolichanthera	56	p5	(TTTCG)3	15	131858	131872
Aspidistra dolichanthera	57	p4	(CATT)3	12	135744	135755
Aspidistra dolichanthera	58	p1	(A)10	10	140100	140109
Aspidistra dolichanthera	59	p2	(TC)5	10	150563	150572
Aspidistra crassifila	1	p1	(A)10	10	2936	2945
Aspidistra crassifila	2	p1	(A)15	15	3556	3570
Aspidistra crassifila	3	p4	(TAAT)3	12	4447	4458
Aspidistra crassifila	4	p1	(A)10	10	4535	4544
Aspidistra crassifila	5	p3	(TTA)4	12	4645	4656
Aspidistra crassifila	6	p1	(C)14	14	4921	4934
Aspidistra crassifila	7	p1	(T)10	10	8172	8181
Aspidistra crassifila	8	p2	(TA)6	12	9206	9217
Aspidistra crassifila	9	p2	(AT)5	10	14649	14658
Aspidistra crassifila	10	p3	(TTA)4	12	15709	15720
Aspidistra crassifila	11	p1	(T)15	15	16465	16479
Aspidistra crassifila	12	p1	(T)10	10	18539	18548
Aspidistra crassifila	13	p1	(T)11	11	18645	18655

ID	SSR nr.	SSR type	SSR	size	start	end
Aspidistra crassifila	14	p2	(AT)5	10	20024	20033
Aspidistra crassifila	15	p1	(A)12	12	22700	22711
Aspidistra crassifila	16	p1	(T)11	11	23093	23103
Aspidistra crassifila	17	p3	(ATA)4	12	30030	30041
Aspidistra crassifila	18	p2	(TA)5	10	30076	30085
Aspidistra crassifila	19	p1	(T)10	10	32208	32217
Aspidistra crassifila	20	p1	(T)10	10	42497	42506
Aspidistra crassifila	21	p1	(A)16	16	44341	44356
Aspidistra crassifila	22	p1	(A)15	15	47098	47112
Aspidistra crassifila	23	p2	(AT)5	10	47178	47187
Aspidistra crassifila	24	p1	(A)12	12	52364	52375
Aspidistra crassifila	25	p1	(T)11	11	55343	55353
Aspidistra crassifila	26	p2	(TA)5	10	55711	55720
Aspidistra crassifila	27	p4	(ATTG)3	12	57603	57614
Aspidistra crassifila	28	c	(AT)5ca(AT)5	22	58016	58037
Aspidistra crassifila	29	p1	(T)10	10	58570	58579
Aspidistra crassifila	30	p2	(TC)5	10	62057	62066
Aspidistra crassifila	31	p4	(AATG)3	12	62676	62687
Aspidistra crassifila	32	p2	(TA)5	10	68668	68677
Aspidistra crassifila	33	p2	(AT)9	18	69119	69136
Aspidistra crassifila	34	p1	(A)11	11	71499	71509
Aspidistra crassifila	35	p1	(T)13	13	71755	71767
Aspidistra crassifila	36	p1	(A)10	10	72524	72533
Aspidistra crassifila	37	p1	(T)10	10	75216	75225
Aspidistra crassifila	38	p1	(T)10	10	83084	83093
Aspidistra crassifila	39	p4	(AATA)3	12	83131	83142
Aspidistra crassifila	40	p1	(T)10	10	83643	83652
Aspidistra crassifila	41	p2	(GA)5	10	90947	90956
Aspidistra crassifila	42	p4	(GAAT)3	12	105753	105764
Aspidistra crassifila	43	p5	(CGAAA)3	15	109637	109651
Aspidistra crassifila	44	p1	(A)10	10	113840	113849
Aspidistra crassifila	45	p1	(A)13	13	114017	114029
Aspidistra crassifila	46	p1	(A)10	10	114157	114166
Aspidistra crassifila	47	p1	(C)12	12	114321	114332
Aspidistra crassifila	48	p1	(T)10	10	114560	114569
Aspidistra crassifila	49	p4	(AATT)3	12	115190	115201
Aspidistra crassifila	50	p1	(T)10	10	115294	115303
Aspidistra crassifila	51	p3	(ATT)4	12	115655	115666
Aspidistra crassifila	52	p1	(A)11	11	116415	116425
Aspidistra crassifila	53	p4	(AATA)3	12	117238	117249
Aspidistra crassifila	54	p2	(TA)5	10	119242	119251
Aspidistra crassifila	55	p4	(TTGA)3	12	119332	119343
Aspidistra crassifila	56	p1	(T)10	10	120408	120417
Aspidistra crassifila	57	p1	(A)10	10	122372	122381
Aspidistra crassifila	58	p3	(ATT)4	12	125132	125143
Aspidistra crassifila	59	p1	(T)10	10	126270	126279
Aspidistra crassifila	60	p1	(A)10	10	128704	128713
Aspidistra crassifila	61	p5	(TTTCG)3	15	131882	131896

ID	SSR nr.	SSR type	SSR	size	start	end
Aspidistra crassifila	62	p4	(CATT)3	12	135768	135779
Aspidistra crassifila	63	p2	(TC)5	10	150577	150586
Aspidistra erecta	1	p3	(CAG)4	12	675	686
Aspidistra erecta	2	p1	(A)10	10	2942	2951
Aspidistra erecta	3	p1	(A)12	12	3563	3574
Aspidistra erecta	4	p4	(TAAT)3	12	4452	4463
Aspidistra erecta	5	p3	(TTA)4	12	4649	4660
Aspidistra erecta	6	p1	(C)12	12	4930	4941
Aspidistra erecta	7	p1	(T)10	10	7708	7717
Aspidistra erecta	8	p1	(T)10	10	8166	8175
Aspidistra erecta	9	p4	(TTAA)3	12	9137	9148
Aspidistra erecta	10	p1	(A)11	11	9486	9496
Aspidistra erecta	11	p1	(T)12	12	13563	13574
Aspidistra erecta	12	p2	(AT)5	10	14598	14607
Aspidistra erecta	13	p3	(TTA)4	12	15658	15669
Aspidistra erecta	14	p1	(T)14	14	16414	16427
Aspidistra erecta	15	p1	(T)10	10	18481	18490
Aspidistra erecta	16	p1	(T)11	11	18587	18597
Aspidistra erecta	17	p2	(AT)5	10	19966	19975
Aspidistra erecta	18	p1	(A)10	10	22648	22657
Aspidistra erecta	19	p1	(T)10	10	23039	23048
Aspidistra erecta	20	p1	(A)11	11	27820	27830
Aspidistra erecta	21	p3	(ATA)4	12	29978	29989
Aspidistra erecta	22	p2	(TA)5	10	30024	30033
Aspidistra erecta	23	p1	(T)10	10	32156	32165
Aspidistra erecta	24	p1	(A)14	14	44305	44318
Aspidistra erecta	25	p1	(A)15	15	47043	47057
Aspidistra erecta	26	p2	(AT)6	12	47123	47134
Aspidistra erecta	27	p1	(T)11	11	48030	48040
Aspidistra erecta	28	p5	(TTGAT)3	15	51524	51538
Aspidistra erecta	29	p1	(A)10	10	52310	52319
Aspidistra erecta	30	p1	(T)10	10	55287	55296
Aspidistra erecta	31	p2	(TA)5	10	55668	55677
Aspidistra erecta	32	p4	(ATTG)3	12	57560	57571
Aspidistra erecta	33	c	(AT)5ca(AT)5	22	57972	57993
Aspidistra erecta	34	p2	(TC)5	10	62002	62011
Aspidistra erecta	35	p2	(TA)5	10	68619	68628
Aspidistra erecta	36	p2	(AT)9	18	69070	69087
Aspidistra erecta	37	p1	(T)11	11	69632	69642
Aspidistra erecta	38	p4	(TAAA)3	12	70331	70342
Aspidistra erecta	39	p1	(A)11	11	71447	71457
Aspidistra erecta	40	p1	(T)11	11	71703	71713
Aspidistra erecta	41	p1	(A)10	10	72503	72512
Aspidistra erecta	42	p1	(T)10	10	75163	75172
Aspidistra erecta	43	p1	(T)10	10	83014	83023
Aspidistra erecta	44	p4	(AATA)3	12	83061	83072
Aspidistra erecta	45	p1	(T)10	10	83768	83777
Aspidistra erecta	46	p2	(GA)5	10	90905	90914

ID	SSR nr.	SSR type	SSR	size	start	end
Aspidistra erecta	47	p1	(T)10	10	101373	101382
Aspidistra erecta	48	p4	(GAAT)3	12	105726	105737
Aspidistra erecta	49	p5	(CGAAA)3	15	109617	109631
Aspidistra erecta	50	p1	(A)11	11	114131	114141
Aspidistra erecta	51	p1	(C)11	11	114296	114306
Aspidistra erecta	52	p4	(AATT)3	12	115163	115174
Aspidistra erecta	53	p3	(ATT)4	12	115627	115638
Aspidistra erecta	54	p1	(A)11	11	116387	116397
Aspidistra erecta	55	p4	(AATA)3	12	117210	117221
Aspidistra erecta	56	p4	(TTGA)3	12	119307	119318
Aspidistra erecta	57	p1	(T)11	11	120383	120393
Aspidistra erecta	58	p1	(A)10	10	122348	122357
Aspidistra erecta	59	p1	(T)10	10	126246	126255
Aspidistra erecta	60	p1	(A)10	10	128683	128692
Aspidistra erecta	61	p5	(TTTCG)3	15	131867	131881
Aspidistra erecta	62	p4	(CATT)3	12	135760	135771
Aspidistra erecta	63	p1	(A)10	10	140116	140125
Aspidistra erecta	64	p2	(TC)5	10	150584	150593
Aspidistra longgangensis	1	p1	(A)11	11	2942	2952
Aspidistra longgangensis	2	p1	(A)16	16	3563	3578
Aspidistra longgangensis	3	p4	(TAAT)3	12	4455	4466
Aspidistra longgangensis	4	p3	(TTA)4	12	4652	4663
Aspidistra longgangensis	5	p1	(C)12	12	4928	4939
Aspidistra longgangensis	6	p1	(A)10	10	8343	8352
Aspidistra longgangensis	7	p2	(TA)8	16	9202	9217
Aspidistra longgangensis	8	p1	(A)10	10	9543	9552
Aspidistra longgangensis	9	p2	(AT)5	10	14650	14659
Aspidistra longgangensis	10	p3	(TTA)4	12	15710	15721
Aspidistra longgangensis	11	p1	(T)15	15	16466	16480
Aspidistra longgangensis	12	p1	(T)10	10	18504	18513
Aspidistra longgangensis	13	p1	(T)11	11	18610	18620
Aspidistra longgangensis	14	p2	(AT)5	10	19989	19998
Aspidistra longgangensis	15	p1	(T)11	11	21274	21284
Aspidistra longgangensis	16	c	(T)10gactttgttcaat(A)12	35	22642	22676
Aspidistra longgangensis	17	p1	(T)11	11	23058	23068
Aspidistra longgangensis	18	p1	(A)10	10	27839	27848
Aspidistra longgangensis	19	p3	(ATA)4	12	29995	30006
Aspidistra longgangensis	20	p2	(TA)5	10	30041	30050
Aspidistra longgangensis	21	p1	(T)10	10	32173	32182
Aspidistra longgangensis	22	p1	(A)13	13	44325	44337
Aspidistra longgangensis	23	p1	(A)17	17	47054	47070
Aspidistra longgangensis	24	p2	(AT)5	10	47136	47145
Aspidistra longgangensis	25	p1	(T)10	10	48041	48050
Aspidistra longgangensis	26	p1	(A)10	10	52304	52313
Aspidistra longgangensis	27	p1	(T)10	10	52480	52489
Aspidistra longgangensis	28	p2	(TA)5	10	55643	55652
Aspidistra longgangensis	29	p4	(ATTG)3	12	57535	57546
Aspidistra longgangensis	30	c	(AT)5ca(AT)5	22	57948	57969

ID	SSR nr.	SSR type	SSR	size	start	end
Aspidistra longgangensis	31	p1	(A)11	11	59623	59633
Aspidistra longgangensis	32	p2	(TC)5	10	62000	62009
Aspidistra longgangensis	33	p4	(AATG)3	12	62619	62630
Aspidistra longgangensis	34	p2	(TA)5	10	68633	68642
Aspidistra longgangensis	35	p2	(AT)10	20	69088	69107
Aspidistra longgangensis	36	p1	(T)10	10	69646	69655
Aspidistra longgangensis	37	p1	(A)10	10	71461	71470
Aspidistra longgangensis	38	p1	(T)10	10	71988	71997
Aspidistra longgangensis	39	p1	(A)10	10	72483	72492
Aspidistra longgangensis	40	p1	(A)10	10	72516	72525
Aspidistra longgangensis	41	p1	(T)10	10	83029	83038
Aspidistra longgangensis	42	p4	(AATA)3	12	83076	83087
Aspidistra longgangensis	43	p2	(GA)5	10	90904	90913
Aspidistra longgangensis	44	p1	(T)10	10	101367	101376
Aspidistra longgangensis	45	p1	(T)11	11	104424	104434
Aspidistra longgangensis	46	p4	(GAAT)3	12	105722	105733
Aspidistra longgangensis	47	p5	(CGAAA)3	15	109599	109613
Aspidistra longgangensis	48	p1	(A)13	13	114115	114127
Aspidistra longgangensis	49	p1	(T)10	10	114517	114526
Aspidistra longgangensis	50	p4	(AATT)3	12	115142	115153
Aspidistra longgangensis	51	p1	(T)10	10	115246	115255
Aspidistra longgangensis	52	p3	(ATT)4	12	115608	115619
Aspidistra longgangensis	53	p1	(A)11	11	116368	116378
Aspidistra longgangensis	54	p4	(AATA)3	12	117191	117202
Aspidistra longgangensis	55	p4	(TTGA)3	12	119285	119296
Aspidistra longgangensis	56	p1	(A)10	10	122315	122324
Aspidistra longgangensis	57	p3	(ATT)4	12	125075	125086
Aspidistra longgangensis	58	p1	(T)10	10	126213	126222
Aspidistra longgangensis	59	p1	(A)10	10	128647	128656
Aspidistra longgangensis	60	p5	(TTTCG)3	15	131825	131839
Aspidistra longgangensis	61	p4	(CATT)3	12	135704	135715
Aspidistra longgangensis	62	p1	(A)11	11	137004	137014
Aspidistra longgangensis	63	p1	(A)10	10	140062	140071
Aspidistra longgangensis	64	p2	(TC)5	10	150525	150534
Aspidistra nankunshanensis	1	p1	(A)11	11	2942	2952
Aspidistra nankunshanensis	2	p1	(A)14	14	3563	3576
Aspidistra nankunshanensis	3	p4	(TAAT)3	12	4453	4464
Aspidistra nankunshanensis	4	p1	(A)10	10	4541	4550
Aspidistra nankunshanensis	5	p3	(TTA)4	12	4651	4662
Aspidistra nankunshanensis	6	p1	(C)13	13	4929	4941
Aspidistra nankunshanensis	7	p1	(T)10	10	6489	6498
Aspidistra nankunshanensis	8	p1	(T)10	10	7977	7986
Aspidistra nankunshanensis	9	p2	(TA)8	16	9190	9205
Aspidistra nankunshanensis	10	p1	(T)10	10	13604	13613
Aspidistra nankunshanensis	11	p2	(AT)5	10	14638	14647
Aspidistra nankunshanensis	12	p3	(TTA)4	12	15698	15709
Aspidistra nankunshanensis	13	p1	(T)14	14	16454	16467
Aspidistra nankunshanensis	14	p1	(T)10	10	18521	18530

ID	SSR nr.	SSR type	SSR	size	start	end
Aspidistra nankunshanensis	15	p1	(T)11	11	18627	18637
Aspidistra nankunshanensis	16	p2	(AT)5	10	20006	20015
Aspidistra nankunshanensis	17	p1	(A)11	11	22682	22692
Aspidistra nankunshanensis	18	p1	(T)10	10	23074	23083
Aspidistra nankunshanensis	19	p1	(A)10	10	27854	27863
Aspidistra nankunshanensis	20	p3	(ATA)4	12	30011	30022
Aspidistra nankunshanensis	21	p2	(TA)5	10	30057	30066
Aspidistra nankunshanensis	22	p1	(T)10	10	32189	32198
Aspidistra nankunshanensis	23	p1	(A)14	14	44372	44385
Aspidistra nankunshanensis	24	p1	(A)13	13	47114	47126
Aspidistra nankunshanensis	25	p2	(AT)5	10	47192	47201
Aspidistra nankunshanensis	26	p1	(T)11	11	48097	48107
Aspidistra nankunshanensis	27	p1	(A)14	14	52380	52393
Aspidistra nankunshanensis	28	p1	(T)10	10	55361	55370
Aspidistra nankunshanensis	29	p2	(TA)5	10	55729	55738
Aspidistra nankunshanensis	30	p4	(ATTG)3	12	57621	57632
Aspidistra nankunshanensis	31	c	(AT)5ca(AT)5	22	58034	58055
Aspidistra nankunshanensis	32	p1	(A)11	11	59709	59719
Aspidistra nankunshanensis	33	p2	(TC)5	10	62059	62068
Aspidistra nankunshanensis	34	p4	(AATG)3	12	62678	62689
Aspidistra nankunshanensis	35	p2	(TA)5	10	68689	68698
Aspidistra nankunshanensis	36	p2	(AT)9	18	69140	69157
Aspidistra nankunshanensis	37	p1	(T)10	10	69707	69716
Aspidistra nankunshanensis	38	p4	(TAAA)3	12	70406	70417
Aspidistra nankunshanensis	39	p1	(A)10	10	71522	71531
Aspidistra nankunshanensis	40	p1	(T)10	10	72049	72058
Aspidistra nankunshanensis	41	p1	(A)11	11	72433	72443
Aspidistra nankunshanensis	42	p1	(A)10	10	72548	72557
Aspidistra nankunshanensis	43	p1	(T)10	10	83108	83117
Aspidistra nankunshanensis	44	p4	(AATA)3	12	83155	83166
Aspidistra nankunshanensis	45	p2	(GA)5	10	90983	90992
Aspidistra nankunshanensis	46	p1	(T)10	10	101464	101473
Aspidistra nankunshanensis	47	p4	(GAAT)3	12	105817	105828
Aspidistra nankunshanensis	48	p5	(CGAAA)3	15	109701	109715
Aspidistra nankunshanensis	49	p1	(A)11	11	114080	114090
Aspidistra nankunshanensis	50	p1	(A)11	11	114218	114228
Aspidistra nankunshanensis	51	p1	(C)10	10	114383	114392
Aspidistra nankunshanensis	52	p1	(T)10	10	114620	114629
Aspidistra nankunshanensis	53	p4	(AATT)3	12	115250	115261
Aspidistra nankunshanensis	54	p3	(ATT)4	12	115714	115725
Aspidistra nankunshanensis	55	p1	(A)11	11	116474	116484
Aspidistra nankunshanensis	56	p4	(AATA)3	12	117297	117308
Aspidistra nankunshanensis	57	p4	(TTGA)3	12	119391	119402
Aspidistra nankunshanensis	58	p1	(T)11	11	120467	120477
Aspidistra nankunshanensis	59	p1	(A)10	10	122432	122441
Aspidistra nankunshanensis	60	p3	(ATT)4	12	125192	125203
Aspidistra nankunshanensis	61	p1	(T)10	10	126330	126339
Aspidistra nankunshanensis	62	p1	(A)10	10	128764	128773

ID	SSR nr.	SSR type	SSR	size	start	end
Aspidistra nankunshanensis	63	p5	(TTTCG)3	15	131942	131956
Aspidistra nankunshanensis	64	p4	(CATT)3	12	135828	135839
Aspidistra nankunshanensis	65	p1	(A)10	10	140184	140193
Aspidistra nankunshanensis	66	p2	(TC)5	10	150665	150674

Note: Two adjacent SSRs separated by less than 20 bp are present in compound formation.
